# A Gender-Bias-Mitigated, Data-Driven Precision Medicine System to Assist in the Selection of Biological Treatments of Grade 3 and 4 Knee Osteoarthritis: Development and Preliminary Validation of precisionKNEE

**DOI:** 10.7759/cureus.55832

**Published:** 2024-03-09

**Authors:** Nima Heidari, Stefano Olgiati, Davide Meloni, James Parkin, Brady Fish, Mark Slevin, Leonard Azamfirei

**Affiliations:** 1 Discovery Driven Precision Medicine, European Quantum Medical, London, GBR; 2 Medical Supercomputation and Biostatistics, European Quantum Medical, Milan, ITA; 3 Supercomputation and Artificial Intelligence, European Quantum Medical, Turin, ITA; 4 Radiology, Norfolk and Norwich University Hospitals National Health Service (NHS) Foundation Trust, London, GBR; 5 Board Member, European Quantum, Philadelphia, USA; 6 Medicine, Pharmacy, Science and Technology, George Emil Palade University, Targu Mures, ROU

**Keywords:** non-surgical treatment, total knee replacement(tkr), artificial intelligence in health care, precision medicine, microfragmented fat injection, orthopaedic biologics, knee osteoarthritis/ koa

## Abstract

Objective

To identify key variables predictive of patient responses to microfragmented adipose tissue (MFAT) treatment in knee osteoarthritis (KOA) and evaluate its potential to delay or mitigate the need for total knee replacement (TKR).

Methods

We utilised a dataset comprising 329 patients treated with MFAT for KOA, incorporating variables such as gender, age, BMI, arthritic aetiology, radiological grade, and Oxford Knee Scores (OKS) pre- and post-treatment. We employed random forest regressors for model training and testing, with gender bias mitigation and outlier detection to enhance prediction accuracy. Model performance was assessed through root mean squared error (RMSE) and mean absolute error (MAE), with further validation in a TKR-suitable patient subset.

Results

The model achieved a test RMSE of 6.72 and an MAE of 5.38, reflecting moderate predictive accuracy across the patient cohort. Stratification by gender revealed no statistically significant differences between actual and predicted OKS improvements (p-values: males = 0.93, females = 0.92). For the subset of patients suitable for TKR, the model presented an increased RMSE of 9.77 and MAE of 7.81, indicating reduced accuracy in this group. The decision tree analysis identified pre-operative OKS, radiological grade, and gender as significant predictors of post-treatment outcomes, with pre-operative OKS being the most critical determinant. Patients with lower pre-operative OKS showed varying responses based on radiological severity and gender, suggesting a nuanced interaction between these factors in determining treatment efficacy.

Conclusion

This study highlights the potential of MFAT as a non-surgical alternative for KOA treatment, emphasising the importance of personalised patient assessments. While promising, the predictive model warrants further refinement and validation with a larger, more diverse dataset to improve its utility in clinical decision-making for KOA management.

## Introduction

Knee osteoarthritis (KOA) ranks as the 11th highest global disability contributor with a 3.8% prevalence (95% UI: 3.6% to 4.1%) [1]. With an ageing demographic and rising obesity, osteoarthritis is predicted to impact 15.7% of the global population by 2032 [2]. In England, 4.11 million adults over 45 have KOA; 6.1% are severely affected. The direct cost of arthritis treatment to the UK healthcare system is around £10.2 billion annually, with lost workdays costing £2.58 billion in 2017, projected to rise to £3.43 billion by 2030. Annually since 2015, over 100,000 total knee replacements (TKR) have been conducted in the UK, but approximately 20% of those receiving TKR report dissatisfaction [3,4]. Although patient satisfaction is crucial for evaluating TKR success, it's underreported and inconsistently measured [5]. Reducing the reliance on TKR is advantageous for both patients and financial stakeholders [6]. Microfragmented adipose tissue (MFAT) shows potential in addressing arthritis pain and enhancing function, proving effective in both early and late KOA stages [7,8]. MFAT is readily acquired from the patient’s own adipose tissues subcutaneously and its micro-fragmentation at the bedside means it can be administered as a single injection into the arthritic knee. Details of this procedure have been previously described in detail [7]. Our prior studies confirmed MFAT's effectiveness in decreasing KOA pain at one year and analysed gender bias impacts [9]. Furthermore, we assessed the benefits in a two-year knee replacement cohort [6].

Our aim was to identify the most relevant variables for predicting patient responses to MFAT. We then applied this to the cohort within our dataset that would be suitable for TKR to assess whether we could predict an improvement in their pain and function following an MFAT injection, thereby delaying or mitigating the need for surgical intervention.

An earlier version of this study was originally uploaded on the medRxiv preprint server [10].

## Materials and methods

Harvesting the adipose tissue and injecting MFAT

Adipose tissue was harvested and microfragmented using a previously published technique [7]. The MFAT was then injected under ultrasonographic guidance into the knee joint. The procedure was performed under sedation in an operating theatre. Following a full recovery, the patients were discharged with a physiotherapy protocol. The Research Ethics Committee of the George Emil Palade University of Medicine, Pharmacy, Science and Technology, Targu Mures, Romania issued approval (No. 1464/2021).

Dataset

The dataset used was generated from the biologic treatment of KOA with MFAT in 142 women and 187 men, correlating to 113980 data points [6,7,9]. Variables considered were gender, age, body mass index, aetiology of arthritic disease, radiological arthritic severity grade using the Kellgren-Lawrence scale [11], pre-operative Oxford Knee Score (OKS) and one-year follow-up OKS.

Oxford Knee Score

OKS comprises 12 questions that are scored 0-4 with 0 being severe compromise and 4 being no compromise, covering pain and function of the knee [12]. The best outcome is a score of 48 and the worst score possible is 0. This is a validated score for the measure of functional outcomes in patients undergoing TKR.

All 329 patients completed the OKS questionnaire before treatment, and at three months, six months and one year following treatment.

Response class

The response was computed by subtracting the preoperative OKS from the one-year follow-up OKS. Positive values indicate an improvement in pain and function with a commensurate increase in the OKS whereas negative values mean a deterioration in the patient's pain and function.

Gender-bias mitigation

Our previous work demonstrates the gender bias present within our dataset [9]. Bias can arise in machine learning at multiple stages, and imbalanced datasets may significantly impair prediction accuracy. Although eliminating bias entirely is unfeasible, minimising it to balance bias and variance is achievable. Employing mitigation strategies can reduce bias impacts and enhance prediction accuracy despite dataset imbalances. The majority class (male patients) was down-sampled to match the minority class (female patients) to address this bias [13]. This conditional data partition balanced our dataset whilst maintaining the class distribution of the dataset (see the Results section).

Outlier detection

Following gender bias mitigation, the dataset contained a total of 286 patients. The dataset was then partitioned into training (n= 231 equivalent to 80%) and test (n= 55 equivalent to 20%) sets whilst ensuring that the “response” variable was evenly distributed within each partition. We then removed the outliers from the training dataset to avoid skewing [14]. Data was labelled as outlying if its response variable value fell more or less than 3 times the median absolute deviation from the median. In this case, 13 outliers were identified These outliers were then removed from the training data, resulting in a cleaned training dataset with 218 observations.

Model training and testing

All variables other than response and one-year follow-up OKS were considered as predictor features for both model training and testing. The test set represents 20% of the size of the balanced dataset, including outliers (n = 55). Training observations were regressed against the absolute response outcome variable utilising random forest regressors [15]. Performance metrics were then produced using the test dataset. Of note, the test dataset included outlying observations to avoid significant data augmentation rendering the test data non-representative of the population data.

Model validation in population suitable for TKR

Models were validated using both the full test dataset and a subset of test data from patients with Kellgren-Lawrence grade 3 and 4 radiological evidence of arthritis, age greater than 64, pre-operative OKS less than or equal to 27 and idiopathic aetiology of arthritis. The group of patients with these criteria would be suitable for TKR [6]. The ability to predict response to an injection of MFAT may help in choosing the best candidates for this less invasive treatment, thereby delaying the need for TKR.

In both cases, a Wilcoxon signed rank test on paired samples between actual and predicted values was performed. These metrics are reported post-stratification by gender (see the Results section).

## Results

Descriptive analysis

Patients in our dataset, prior to gender bias mitigation, had a mean age of 66.4 years on the date of the procedure with a standard deviation of 10.2 years. The mean BMI of our patient cohort was 26.9 with an associated standard deviation of 4.3. A gender imbalance with 187 (56.8%) male patients and 142 (43.2%) female patients was present in our dataset.

The majority 90.6% (n = 298) of patients had idiopathic arthritis, a further 6.7% (n = 22) of patients had trauma-related arthritis and 2.7% (n = 9) of patients had inflammatory arthritis. 53.5% (n = 176) of patients had grade 4 radiological evidence of arthritis and 46.5% (n = 153) had grade 3 or better radiological evidence of arthritis. The mean pre-operative OKS was 31.6 with an associated standard deviation of 8.7. Table 1 outlines our exploratory analysis prior to, and following gender bias mitigation.

**Table 1:** Patients’ characteristics before and after gender-bias mitigation by randomised under-sampling. IQR: interquartile range, OA: osteoarthritis, OKS: Oxford Knee Score, SD: standard deviation.

		Prior to bias mitigation	After bias mitigation
Gender	Female	142	142
	Male	187	144
Age	Range	32 - 90	32 - 90
	Mean (SD)	66.4 (10.2)	66.7 (10.1)
BMI	Range	16 - 42	16 - 42
	Median (IQR)	26 (24 - 30)	26 (24 - 30)
OA grade (Number (%))	0	1 (0)	1 (0)
	1	18 (5)	15 (5)
	2	59 (18)	54 (19)
	3	75 (23)	66 (23)
	4	176 (53)	152 (52)
Pre-op OKS	Range	1 - 48	1 - 48
	Median (IQR)	32 (26 – 39)	32 (26 – 38)
Post-op OKS	Range	5 - 48	5 - 48
	Median (IQR)	39 (31 – 45)	38 (31 – 44)
Response	Range	-17 – 34	-17 – 34
	Median (IQR)	5 (-1 – 11)	5 (-1 – 11)

Correlation matrix of features and OKS at one year

**Figure 1:**
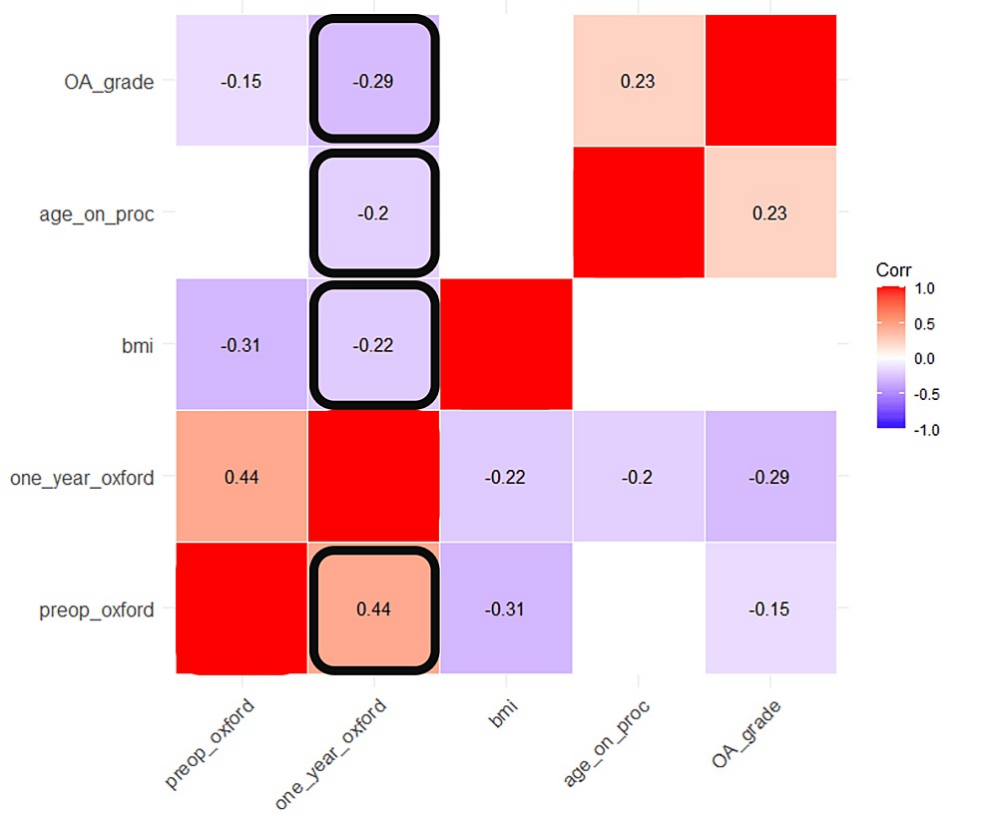
Correlation matrix showing the Pearson correlation coefficients for continuous features. Statistically insignificant coefficients have been blanked out. A black square has been drawn to define the important correlations between the preoperative variables and the one-year OKS. OKS: Oxford Knee Score.

The Pearson correlation coefficient was calculated for each continuous feature against every other continuous feature with the most relevant correlation being between each of the features and the outcome, the one-year post-procedure OKS. Figure 1 visualises this relationship in the form of a correlation matrix. We note that the strongest statistically significant linear correlation observed was between pre-operative OKS and one-year OKS at a coefficient of 0.44. Age at procedure (-0.2), BMI (-0.22) and radiological grade of arthritis (-0.29) were all negatively associated with OKS at one year.

Model results

Our model was trained without outliers using the cleaned dataset. The model was then tested on data without outlier removal. Test Root Mean Squared Error (RMSE) was 6.72 and the Mean Absolute Error (MAE) was 5.38. The MAE measures the average magnitude of errors in the predictions. An MAE of 5.38 means that, on average, the model's predictions are 5.38 units away from the actual values. Although similar to the RMSE, MAE gives a clearer picture of the model's predictive accuracy as it's not as sensitive to outliers as RMSE.

**Figure 2:**
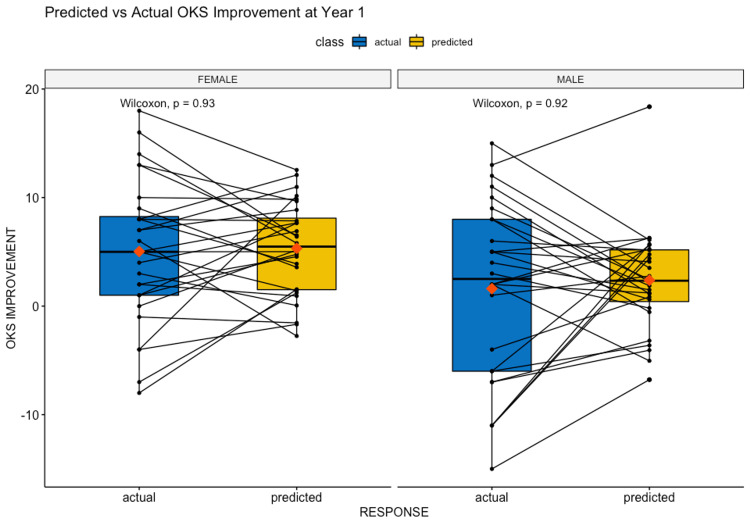
Predicted vs. actual OKS values; with Wilcoxon significance levels. OKS: Oxford Knee Score.

The final model’s predictions were compared with the actual OKS improvement using Wilcoxon signed-rank tests. Male and female gender-stratified predictions had p-values of 0.93 and 0.92 respectively, providing us with no evidence of statistically significant differences in sample medians between actual and predicted values. Figure 2 visualises this result.

Model validation in population suitable for TKR

Further testing of the model with the subpopulation of 45 patients suitable for TKR (as outlined in methods) demonstrated an RMSE of 9.77 and an MAE of 7.81. Values demonstrated here when compared to the model testing undertaken without outlier removal, noted a decrease in the model’s predictive accuracy with the commensurate increase in the RMSE and MAE.

**Figure 3:**
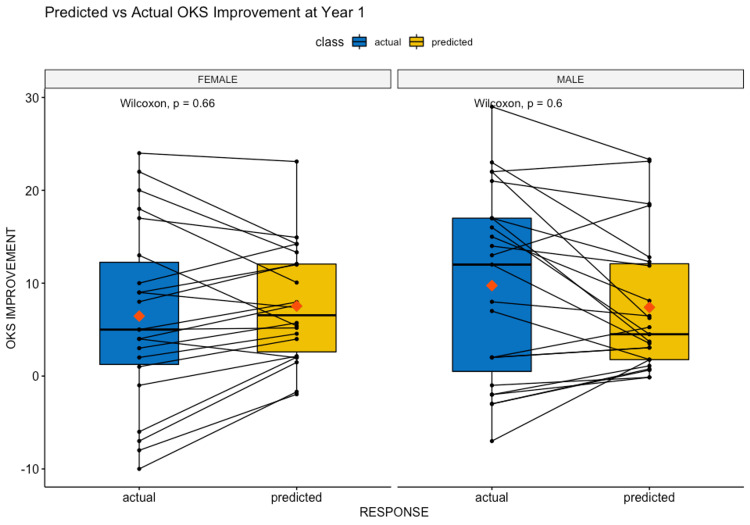
Predicted vs. actual OKS values filtered by suitability for TKR. OKS: Oxford Knee Score, TKR: total knee replacement.

Figure 3 demonstrates the actual vs predicted results of our final model using this subset data for testing. compared with the actual OKS improvement using Wilcoxon signed-rank tests. Male and female gender-stratified predictions had p-values of 0.6 and 0.66 respectively, demonstrating no evidence of statistically significant differences in sample medians between actual and predicted values.

Model interpretation

**Figure 4:**
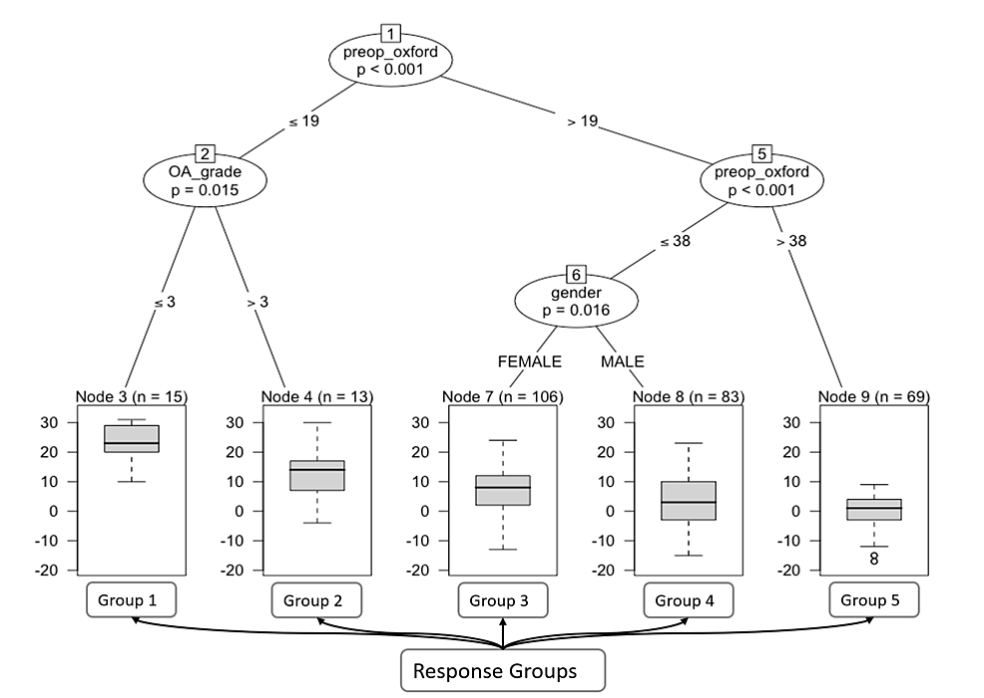
Conditional inference tree analysis of preoperative and demographic factors influencing postoperative outcomes. Each terminal node (Group 1-5) is associated with a boxplot that visualizes the distribution of the post-operative improvement in OKS, or response. The boxplots include the median (central line), interquartile range (box edges), and potential outliers (individual points), providing a summary of the outcome distribution for patients within each subgroup. Statistical significance is denoted at each decision point, with p-values indicating the likelihood that the observed distribution of outcomes is due to chance. Lower p-values (< 0.05) suggest a stronger statistical justification for the chosen splits based on the respective variables. OKS: Oxford Knee Score.

To interpret and explain our results, a conditional inference tree was computed outlining the most significant features our models identified. Figure 4 demonstrates three major features contributing to an observation being classified into one of five response groups.

This decision tree illustrates the hierarchical relationship between preoperative functional status as measured by the OKS (preop_Oxford), radiological arthritis severity score (OA_grade), and gender, in determining postoperative outcomes. Each node represents a decision point that splits the dataset based on the value of a predictor variable, guided by statistically significant thresholds.

We found pre-operative OKS to be the most important feature. The initial decision node (Node 1) divides the patient population based on this, with a cut-off value of 19. A lower score indicates a more severe impairment in function before surgery.

If the preoperative OKS is ≤19, the tree progresses to Node 2, which considers the Kellgren-Lawrence radiographic severity of OA. At this node, the OA grade ≤3 leads to Node 3 (Response Group 1), while an OA grade >3 leads to Node 4 (Response Group 2). Group 1 and 2 consists of 15 and 13 patients respectively and represent patients with lower preoperative function, segregated by OA severity. Group 1, which includes patients with milder OA (grade ≤3), shows a different outcome distribution compared to Group, which encompasses patients with more severe OA (grade >3)

Node 5 serves as a secondary decision point for patients with better pre-operative functional status. A score of ≤38 on the preoperative OKS prompts an evaluation based on gender (Node 6), resulting in Node 7 (Response Group 3) for females and Node 8 (Response Group 4) for males. Thus, differentiating the outcomes for females and males with moderate preoperative function (preoperative OKS ≤38), respectively.

Patients with a preoperative OKS >38 are directed to Node 9 (Group 5), indicating a relatively high preoperative functional status irrespective of OA grade or gender. This group show the least improvement in their postoperative OKS at one year. The most likely reason for this is due to the ceiling effect of the OKS. The highest score is 48 and as the patients reach the higher score further improvement becomes more difficult to measure with this instrument.

## Discussion

The study's findings underscore the potential of MFAT injections as a viable non-surgical treatment for KOA, particularly in patients suitable for TKR. By identifying key variables that predict patient responses to MFAT, such as pre-operative OKS, radiological grade of arthritis, and gender, the study advances our understanding of personalised treatment approaches for KOA. The mitigation of gender bias in the dataset and the inclusion of a wide range of patient demographics enhance the robustness and applicability of the predictive model.

However, the model's performance, as indicated by the RMSE and MAE, suggests variability in the predictive accuracy. While the model shows promise in approximating patient outcomes post-MFAT treatment, the differences in prediction accuracy between the general cohort and the subset suitable for TKR highlight the complexity of predicting treatment outcomes in more advanced stages of KOA. The decision tree analysis details the multifaceted nature of treatment response, emphasising the importance of pre-operative functional status, radiological severity, and gender in determining postoperative outcomes. Other studies have also looked at predictive models of outcomes following MFAT injection [16].

The use of the precisionKNEE algorithm requires external validation with an appropriate study [10]. We believe it needs to be viewed as a diagnostic tool, much like imaging, providing additional information for the clinician and the patient to navigate the complex array of treatments available. Further studies are required to assess precisionKNEE in a clinical setting. Such a study should incorporate other features representing two- or three-dimensional imaging as our research indicates these are important in determining response to biological treatment. The SPIRIT-AI (Standard Protocol Items: Recommendations for Interventional Trials-Artificial Intelligence) and CONSORT-AI (Consolidated Standards of Reporting Trials-Artificial Intelligence) initiatives provides guidance to improve the transparency and completeness of reporting of clinical trials evaluating interventions involving artificial intelligence [17]. As such any study needs to be designed with these in mind.

Limitations

Despite being one of the largest datasets of its kind, upon stratification we observe groups of patients with minimal observations and thus we cannot conclude external validity in the findings pertaining to these groups.

Kellgren-Lawrence classification of arthritis was found to be an important feature during modelling. This measure is crude and its relationship to patient symptoms remains.

MFAT is the only treatment used in our model. With the inclusion of other modalities of treatment such as other biologics including bone marrow aspirate concentrate, platelet-rich plasma, nSTRIDE (Zimmer Biomet, Warsaw, Indiana) as well as TKR, a true clinical decision-making support tool can be developed. This may be viewed by clinicians as an incursion into their territory of decision-making.

## Conclusions

This study contributes valuable insights into the predictive factors for successful microfragmented adipose tissue (MFAT) treatment in knee osteoarthritis (KOA) patients, potentially offering a non-surgical alternative to those considering total knee replacement (TKR). The analysis not only highlights the importance of individualized patient assessment but also underscores the necessity for further research to refine predictive models for KOA treatment outcomes. Future studies should focus on expanding the dataset, incorporating longitudinal follow-up data, and exploring additional variables that may influence treatment effectiveness such as two or three-dimensional imaging as our research indicates these are important in determining response to biological treatment. The SPIRIT-AI (Standard Protocol Items: Recommendations for Interventional Trials-Artificial Intelligence) and CONSORT-AI (Consolidated Standards of Reporting Trials-Artificial Intelligence) initiatives provide guidance to improve the transparency and completeness of reporting of clinical trials evaluating interventions involving artificial intelligence as such any study needs to be designed with these in mind.

Ultimately, enhancing predictive accuracy can lead to more targeted and effective non-surgical interventions for KOA, reducing the reliance on TKR and improving patient satisfaction and functional outcomes.
